# Structural Classification of Wild Boar (*Sus scrofa*) Vocalizations

**DOI:** 10.1111/eth.12472

**Published:** 2016-02-16

**Authors:** Maxime Garcia, Bruno Gingras, Daniel L. Bowling, Christian T. Herbst, Markus Boeckle, Yann Locatelli, W. Tecumseh Fitch

**Affiliations:** ^1^ Department of Cognitive BiologyUniversity of ViennaViennaAustria; ^2^ Voice Research LabDepartment of BiophysicsFaculty of SciencePalacký UniversityOlomoucCzech Republic; ^3^ Department of Psychotherapy and Biopsychosocial HealthDanube University KremsKremsAustria; ^4^ Réserve de la Haute ToucheMuséum National d'Histoire NaturelleObterreFrance; ^5^ Equipe Interactions Cellulaires et FertilitéUMR0085 Physiologie de la Reproduction et des ComportementsInstitut National de la Recherche AgronomiqueNouzillyFrance

**Keywords:** acoustic communication, graded vocalizations, *sus scrofa*, vocal repertoire, wild boar

## Abstract

Determining whether a species' vocal communication system is graded or discrete requires definition of its vocal repertoire. In this context, research on domestic pig (*Sus scrofa domesticus*) vocalizations, for example, has led to significant advances in our understanding of communicative functions. Despite their close relation to domestic pigs, little is known about wild boar (*Sus scrofa)* vocalizations. The few existing studies, conducted in the 1970s, relied on visual inspections of spectrograms to quantify acoustic parameters and lacked statistical analysis. Here, we use objective signal processing techniques and advanced statistical approaches to classify 616 calls recorded from semi‐free ranging animals. Based on four spectral and temporal acoustic parameters—quartile Q25, duration, spectral flux, and spectral flatness—extracted from a multivariate analysis, we refine and extend the conclusions drawn from previous work and present a statistically validated classification of the wild boar vocal repertoire into four call types: grunts, grunt‐squeals, squeals, and trumpets. While the majority of calls could be sorted into these categories using objective criteria, we also found evidence supporting a graded interpretation of some wild boar vocalizations as acoustically continuous, with the extremes representing discrete call types. The use of objective criteria based on modern techniques and statistics in respect to acoustic continuity advances our understanding of vocal variation. Integrating our findings with recent studies on domestic pig vocal behavior and emotions, we emphasize the importance of grunt‐squeals for acoustic approaches to animal welfare and underline the need of further research investigating the role of domestication on animal vocal communication.

## Introduction

A central goal of animal communication research is to understand the function of vocalizations within a species (Bradbury & Vehrencamp 1998) and/or between species (e.g., Zuberbühler 2000). In this context, whether a communication system is better characterized as graded or discrete is a fundamental question (Marler 1975; Morton 1982; Cheney & Seyfarth 1990; Fitch 2010). While graded systems show continuous variation in acoustic structure, lacking strict boundaries between call types, discrete systems show acoustically distinct call types generally lacking structurally intermediate forms (Marler 1975, 1977; Keenan et al. 2013). To investigate this issue, a key step is to define a representative acoustic repertoire of the study species (Hauser 1997). Many studies, especially on primates, have defined vocal repertoires and explored this topic, documenting both graded (Rowell & Hinde 1962; Marler 1976; Fischer & Hammerschmidt 2002) and discrete (Zuberbühler et al. 1997; Arnold & Zuberbühler 2008) repertoires. Some mixed cases have also been reported and showed varying levels of gradation depending on call types and/or sex (Bouchet et al. 2010; Lemasson & Hausberger 2011; Keenan et al. 2013).

Acoustic continuity has also been investigated in non‐primate mammalian species vocalizations (Volodina 2000; Boisseau 2005; Stoeger‐Horwath et al. 2007; Nair et al. 2009). For example, the first description of the domestic pig (*Sus scrofa domesticus*) vocal repertoire by Kiley (1972), identified a degree of acoustic gradation in their definition of ‘grunt‐squeals’: an intermediate vocalization between ‘grunts’ and ‘squeals’. More recent work revisited the pig repertoire (Tallet et al. 2013), similarly concluding that gradation between the acoustic categories is prominent. However, most of the work carried out on domestic pig has focused on a single call type and specific conditions, thus neglecting much of the potential acoustic variability in this species. One reason is that the domestic pig is among the most intensively farmed species on the planet, and studies often focus on specific circumstances (i.e., those particularly relevant to welfare; Whittemore & Kyriazakis 2006). Examples of such focused studies include castration (Puppe et al. 2005), mother‐offspring recognition (Illmann et al. 2002, 2008), nursing (Algers 1993), experimentally‐induced stress (Marchant et al. 2001), or discomfort (Hillmann et al. 2004). This approach has been productive in that it has improved our understanding of how vocalizations reflect the physiological and emotional status of pigs (Schrader & Todt 1998; Düpjan et al. 2008) as well as their interindividual interactions (Kiley 1972; Schön et al. 1999; Melisova et al. 2014). It has also stimulated better assessment practices for housing conditions and overall treatment (Weary et al. 1998; Manteuffel et al. 2004; Puppe et al. 2005; Leidig et al. 2009).

Much less is known about the acoustic signals of the domestic pig's close relative and presumed wild forebear, the wild boar (*Sus scrofa*), from which *domesticus* traces its ancestry (Rothschild & Ruvinsky 2011). Wild and domestic forms remain closely related: hybridization events occur under natural conditions (Scandura et al. 2011), and a given pair of domestic and wild animals will not necessarily be more genetically divergent than two wild animals drawn from geographically distinct populations (Scandura et al. 2008).

Given this close relationship, the investigation of vocal communication in wild boars is needed to establish a comparative foundation for understanding the evolutionary origins and the potential effect of domestication on domestic pig vocalizations. Nonetheless, the vocal repertoire of wild boars has received limited attention, with the only existing publications dating from the 1970s (Klingholz & Meynhardt 1979; Klingholz et al. 1979). These studies were based on data gathered over several years in the wild by ethologist Heinz Meynhardt and colleagues. They classified boar vocalizations into ten call types split between three main groups: voiced sounds or grunts (including contact), unvoiced sounds or squeals/screams (including fear, complaint, defense, fight, isolation, and hunger calls), and intermediate sounds (including alert, alarm, and advertisement calls) (Klingholz et al. 1979). Even though this classification implies discrete call types, in a follow‐up paper these authors discussed acoustic gradation between call types, concluding that although many aspects of wild boar vocalization are continuous, there are also acoustic invariants that may identify calls as discrete entities (Klingholz & Meynhardt 1979). Despite the impressive expertise and understanding these authors developed, their conclusions are potentially limited by a number of factors. First, they relied on visual inspection of spectrograms to define acoustic parameters, which may not provide an objective and consistent measurement method. Second, they performed no statistical analyses on their data. And third, because some of these call types were defined by a single utterance and/or were produced by a single individual, generalizations to the population level may be questionable.

The aim of the current study was to revisit the wild boar vocal repertoire based on the work of Klingholz et al. (1979), taking advantage of advances in digital sound recording and analysis, as well as modern statistical methodologies used in classification designs (Boisseau 2005; Bouchet et al. 2010; Gingras & Fitch 2013; Tallet et al. 2013; Baotic et al. 2014). Our goal was to provide an objective description of the wild boar vocal repertoire and call types, with consideration of acoustical intermediates, as suggested by research on domestic pig vocalization (Kiley 1972). We also applied a more cautious approach to the call type evaluation, which avoids assumptions about the potential meaning of calls. Thus, following the lead of some recent work with domestic pigs (Tallet et al. 2013), we did not assign call types based on behavioral contexts. Rather, we took an acoustic/analytical approach, using multinomial logistic regression modeling and hierarchical cluster analysis of objectively defined acoustic parameters to evaluate a perceptual classification based on the one developed by Klingholz et al. (1979). With this work, we thus establish an acoustically based classification of wild boar vocalizations and provide a basis for further research comparing wild boar and pig vocal behavior. We examine our results in the context of acoustic continuity in animal vocalizations and discuss potential contributions of this study to animal welfare and our understanding of the relationship between domestication processes and vocal communication.

## Material and Methods

### Study Site and Animals

Recordings analyzed in this study were made during spring 2014 (from late February to mid‐May) on the property of a wild boar breeder located in Urciers (46.54°N, 2.13°E, 280 m elevation), France (Société Eric Pradat, hereafter EP), associated with the La Haute‐Touche animal reserve (which belongs to the French Museum National d'Histoire Naturelle). EP provided the first author with full access to wild boar keeping facilities. The largest enclosure at EP (hereafter EP1) measures 110 000 m² and is composed of mixed deciduous forest, a plateau of grassland and bush, and an area with a feeder where food was provided *ad libitum*. EP1 animals' only exposure to humans was a single capture/tagging event (at the age of approximately 3 mo). The EP1 animals thus lived in semi‐free ranging conditions with minimal human contact. This EP1 wild boar group was composed of 3 old adult males and 6 old adult females more than one and a half years old (all visually estimated to weigh between 80 kg and 150 kg by E. Pradat, based on years of experience capturing and selling wild boars to game parks), approximately 55 other young adults of approximately 1 yr in age (all estimated to weigh between 45 kg and 65 kg by E. Pradat; gender unspecified) as well as between 30 and 50 newborn piglets (the number increased during the season). In early May 2014, 19 individuals (9 from EP1 and 10 from another park owned by the same breeder) were captured and placed together in a 30 m² indoor pen for 3 d before being sold to a game park. In both the EP1 and the indoor pen, the population sex ratio was approximately 50% males and 50% females (E. Pradat, personal communication). Finally, 8 1‐yr‐old females were kept in another small enclosure of approximately 300 m² (hereafter EP2) for 6 d, before being transferred to La Haute‐Touche. Recordings were made at all 3 locations (EP1, the indoor pen; and EP2).

### Data Collection

Recordings were made with a Sennheiser ME‐67 directional microphone (frequency response 40‐20000 Hz ± 2.5 dB; Sennheiser Electronic GmbH & Co. KG, Wedemark, Germany) powered by a LR6 battery, connected to a Zoom H4N digital recorder (48 kHz sampling frequency and 16‐bit quantization; Zoom Corporation, Tokyo, Japan); these recordings were stored as uncompressed WAV files. For shock and wind‐noise reduction, the microphone was mounted on a Rycote Modular Windshield WS 7 Kit for Shotgun Microphones, which includes a windshield with suspension system and a synthetic fur windjammer (Rycote Microphone Windshields Ltd, Stroud, UK).

At EP2, recording sessions were made from locations around the edges of the enclosures, mostly at dawn and dusk. At the much larger EP1, recordings were made day and night, whenever individuals showed up to the feeder, mostly from inside the first author's car parked 15–25 m away from the feeder, with the microphone protruding from the window and directed toward the group. In these two outdoor locations, the microphone was positioned 5–40 m from the source individual, whenever the weather conditions permitted a high recording quality. In the indoor pen, recordings were made at a distance of 1–5 m, mostly between 22:00 and 6:00 (in darkness), with the microphone protruding into the pen above the enclosure wall.

Both in the indoor pen and at the outdoor locations (EP1 and EP2), the main obstacle to recording the animals arose from the fact that they were most active at night. Accordingly, the majority of recordings were obtained at night, when lighting conditions made it impossible to clearly identify individual animals. Even under these conditions, however, it was possible to roughly observe size and thus retrieve some information regarding the approximate age of animals (i.e., 1‐yr‐olds were typically smaller than older adults). While recording during the daytime, it was sometimes (in 17% of the cases) possible to determine the sex of the animals (through visualization of testes and/or penile brush)**,** although the identification of specific individuals remained difficult due to the large group size and the rapid succession of interactions. Accordingly, individual identity of the signalers was not considered in this study.

Whenever possible, the type of interaction and/or context in which a vocalization was produced was noted. Unlike individual identification, interaction type and context were easier to assess, as observations of movement, call loudness, and/or the sequence in which calls were emitted did not depend as much on lighting. Using these types of observations and considering the existing literature (McGlone 1985; Meynhardt 1990; Oczak et al. 2013), vocal behavior was sorted into seven classes: (1) alarm, (2) alert/nervous, (3) attacked, (4) chased, (5) contact, (6) scared/threatened, and (7) submissive. When the reaction of the whole group was to flee after a call was produced, this vocalization was contextually labeled as an ‘alarm’ call. Similarly, a call that caused the group to stop its ongoing activity and freeze for a moment was labeled ‘alert/nervous’ call; calls produced when individuals were being physically attacked, typically by a larger individual, were labeled ‘attacked’ calls; vocalizations characterized by a particular call‐sequence produced by individuals being chased by another group member were labeled ‘chased’ calls; calls produced in apparently neutral situations, when animals were foraging at a short distance from each other and/or when they were at rest were labeled ‘contact’ calls; calls produced by individuals being threatened by larger individuals but which managed to escape physical contact were labeled ‘scared/threatened’ calls; finally, calls produced by individuals being threatened by larger individuals but which would initiate a snout‐to‐head contact with the larger individual (around the jaw/cheek area, instead of escape) were labeled ‘submissive’ calls.

### Data Analysis

#### Extraction of acoustic parameters

**Figure 1:**
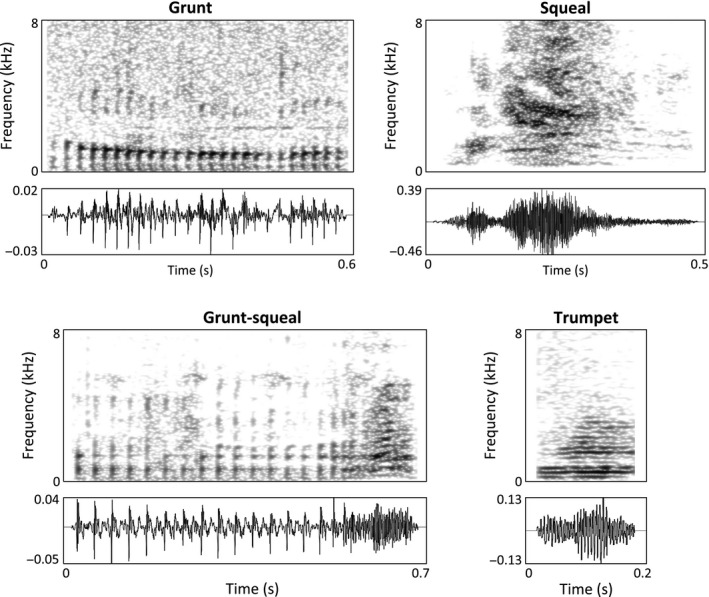
Narrow‐band spectrograms and acoustic waveforms of the four call types identified from our recordings: grunt, squeal, grunt‐squeal, and trumpet. The spectrograms were generated in Praat 5.4.01 using the following settings: Gaussian window shape; time steps: 1000; frequency steps: 250; frequency range: 0–8000 Hz; window length: 0.015 s; dynamic range: 40 dB.

Sound files were initially inspected for quality, and vocalizations produced in the presence of background noise such as wind or anthropogenic activities, other group members running or chewing loudly, other species’ sounds (chorusing frogs, singing birds), or excessive echo (in the case of the recordings made in the indoor pen) were not analyzed. Sound files of acceptable recording quality were retained and annotated with basic call categories (Fig. 1), sex/age and behavioral context using the ‘Annotate: To TextGrid’ function within Praat (Boersma & Weenink 2014). This process led to the selection of 746 high‐quality calls suitable for spectral analysis.

Following descriptions made in previous studies (Kiley 1972; Klingholz et al. 1979; Schrader & Todt 1998), an initial rough perceptual classification of these calls was conducted by author MG after visual inspection of the spectrograms while listening to the recordings. This resulted in the initial identification of five acoustically based categories of calls: grunts, squeals, grunt‐squeals, barks, and trumpets—see all but barks in Fig. 1 (barks were eventually removed from the dataset in this study—see below for further details).

#### Basic call categories

Klingholz et al. (1979) divided wild boar vocalizations into ten call types distributed over three categories (see Introduction). While relying on these three categories to assess the calls recorded in this study, we deemed their subdivision into ten call types too subjective to be followed consistently. For example, what they defined as ‘voiced’ and ‘unvoiced’ sounds (or, respectively, grunts and squeals/screams) are perceptually very distinct, while the perceptual distinction between adult ‘defense’, ‘fight’, and ‘hunger’ calls (all considered as different subtypes of the ‘unvoiced’ category) was ambiguous.

Grunts (see Fig. 1) are pulsatile, low‐frequency sounds whose main spectral energy is below 2 kHz. The time delay between pulses generally suggests fundamental frequencies well below 100 Hz, with rare maxima up to 150 Hz. As such, grunts essentially correspond to the ‘common grunt’ described by Kiley (1972), indicating that ‘the fry are well represented and largely spaced’ (where ‘fry’ is used to indicate the individual pulses of the sound).

Squeals (see Fig. 1) contain energy in a broader frequency range (contrary to grunts, some energy is clearly visible up to 8 kHz). They typically lack the pulsed structure described above, which makes grunts and squeals perceptually highly distinctive. Most often, squeals sound noisy or harsh, although they can also vary in structure and sometimes contain nearly periodic segments. The broad acoustic definition we use to describe squeals includes multiple contextual call types described by Klingholz et al. (1979; namely ‘fear’, ‘complaint’, ‘defense’, ‘fight’, ‘isolation’, and ‘hunger’ calls); again we avoided any assessment relying mainly on behavioral context at this stage.

Cases where both a grunt and a squeal were observed within a single call led us to define an intermediate call category: grunt‐squeals (Kiley 1972 **;** see Fig. 1) are defined here as a mixture between grunts and squeals with both a pulsatile structure and broadband energy, sometimes being a temporal concatenation of a grunt and a squeal with a progressive transition from one to the other, and other times sounding as if both a grunt and a squeal are being produced at the same time.

Barks (Kiley 1972) were isolated, short, high‐intensity vocalizations that are generally non‐harmonic and are usually produced with an abrupt onset, making them harder to discriminate from squeals.

Finally, trumpets (see Fig. 1) are harmonic calls, with a high fundamental frequency (ranging from 200 to 400 kHz and generally lacking energy above 5 kHz) that, contrary to other call types, gave the impression of being produced nasally and with very low intensity (almost impossible to record in EP1 and EP2 as animals were not close enough to the microphone; almost all trumpets analyzed here were recorded in the indoor pen). Of the five call types that were defined in this study, the trumpet was the only type that did not easily fit into the earlier classification based on the intensity, frequency (Klingholz et al. 1979), and ‘tonality’ (Kiley 1972) information provided in previous research.

Nineteen acoustic parameters were extracted from each call included in our analysis (see parameter definitions in the Electronic Supplementary Material—hereafter—Table S1). Call duration (DUR) and spectral energy quartiles (Q25, Q50 and Q75) were extracted using a custom‐built Praat script written by David Reby. The remainder of the acoustical analysis was conducted using the MIR Toolbox 1.4.1 (Lartillot et al. 2008) in MATLAB (V. 7.11.0.284, R2010b, The MathWorks, Inc., Natick, MA, USA), using an analysis window of 1024 samples (~21 ms at a sampling rate of 48000 Hz) with a 50% overlap between adjacent analysis windows. Each consecutive portion of the acoustic signal was multiplied by a Hamming window before calculating a 1024‐point discrete Fourier transform. The following parameters were extracted for each analysis window: mean dominant frequency (DF), spectral centroid (SC), spectral entropy (ENT), coefficient of variation of the root‐mean‐square of the amplitude (CVA), spectral flatness (FLAT), spectral flux (SF), and zero crossing rate (ZC). For several parameters, the following additional features were calculated: means of the differentials between consecutive window frames, which provide information regarding the positive or negative trend of a parameter over the duration of a vocalization; maximum differentials, which provide information regarding the rate of change of a parameter; and standard deviations, which provide information regarding the overall variation within a parameter. This resulted in the extraction of the following parameters: mean differential of the dominant frequency (DDF), maximum differential of the dominant frequency (MDDF), standard deviation of the dominant frequency (STDF), mean differential of the normalized root‐mean‐square (DRMS), standard deviation of the normalized root‐mean‐square (STRMS), mean differential of the spectral flux (DSF), maximum differential of the spectral flux (MDSF), and standard deviation of the spectral flux (STSF).

Other frequently examined acoustic parameters, such as fundamental frequency or formants (as well as related parameters, such as the harmonics‐to‐noise ratio [HNR] or Mel‐Frequency Cepstral Coefficients [MFCC]) were not included because they could not be accurately measured in all vocalizations.

#### Selection of the sample

As the acoustic components of a vocalization depend on the physical characteristics of both the sound source and the vocal tract, it is likely that the parameters extracted here vary as a function of the age (because older adults were larger and thus are expected to have longer vocal tracts and vocal folds). Accordingly, variability introduced by combining calls from older adults with calls from 1‐yr‐old adults could potentially prevent the identification of call types on the basis of acoustic parameters. To circumvent this issue and to use comparable cases, vocalizations from older adults were excluded, given that calls produced by 1‐yr‐old adults represented the main part of the data set (642 calls out of 746).

To establish a representative sample of the group's vocal repertoire, calls had to be collected from as many individuals as possible. Experience gathered from daily observations indicated that animals came to the feeder location in relatively large groups (often above 15, up to approximately 70 individuals) and that at these times, most animals would vocalize. Furthermore, although individual identification could not be performed during recordings at night, behavior at the feeder location typically involved high numbers of interactions and calls coming from diverse spatial locations (with respect to the experimenter, MG), indicating the presence of multiple vocalizing individuals. Together, these facts suggest that the recordings analyzed are representative of the target group's vocal repertoire.

A random sample of ten calls from each call type was selected for a blind evaluation (i.e., disregarding any information on the behavioral context in which they were produced) to determine whether knowledge of the context influenced call type classification. Following this procedure, it was found that barks could not be reliably classified without knowledge of the behavioral context, and upon re‐listening barks were often classified as other call types (grunts, squeals, or grunt‐squeals). Consequently, the 26 bark recordings were also removed from the data set, leaving a total of 616 calls to be used for further analysis**.** Of these 616 calls, the context could be determined in 96% of the cases**.** In all cases**,** calls were from individuals of the same age class, from which approximately 2% were females, 14% were males and 83% were unspecified gender.

### Statistical Analysis

To assess the validity of the initial perceptual classification, made after listening to the recordings and visually inspecting the sound spectrograms, two series of statistical validations were run. First, a multinomial logistic regression was performed to identify the set of parameters that best fit the perceptual classification. Based on the selected parameters, a hierarchical cluster analysis was conducted, the output of which was compared with the perceptual classification. Statistical analyses were conducted in SPSS Statistics (V. 21.0) and in MATLAB. Two‐tailed p‐values are reported and significance levels were set at 0.05.

The statistical distribution for each of the 19 extracted parameters was tested for normality using Shapiro–Wilk tests in conjunction with inspection of Q–Q plots. Significant deviations from normality were observed for all parameters (all ps < 0.05). This prevented use of a discriminant function analysis to test the validity of the perceptual classification, and suggested the use of a Multinomial Logistic Regression (MLR) model instead.

Prior to running the MLR models, all parameters were normalized to z‐scores to obtain unit‐free parameters, and data were weighted according to frequency to avoid biases that could have been introduced by the fact that each call type class included a different number of cases. Data weighting was performed following a uniform prior distribution that stipulated an equal prior probability for all four call types. This prevented the MLR algorithm fitting the data with an equation that mostly depended on the most frequent call types. The multicollinearity of the 19 measured variables was also assessed by computing a Pearson's correlation matrix. For each pair of parameters with a correlation higher than 0.7, one of the two parameters was removed. The choice of the parameter to remove was determined by: (1) whether a parameter was derived from another feature, in which case the derived parameter was removed (e.g., mean spectral flux was selected over standard deviation of the spectral flux); this step led to the exclusion of STDF, STSF, and MDSF; and (2) which parameter had higher correlations with other variables, in which case the parameter with the higher number of correlations was excluded (this step led to the exclusion of Q50, Q75, ZC, SC, and ENT). This procedure thus resulted in 11 parameters being retained for the MLR: DUR, Q25, DF, DDF, MDDF, CVA, DRMS, STRMS, FLAT, SF, and DSF. In this group, one correlation >0.7 remained between DF and Q25. As no obvious reason led to the choice of one of these parameters over the other, two different MLR models including 10 parameters were run, one with DF but excluding Q25 (DF regression) and one with Q25 but excluding DF (Q25 regression). The MLR models used forward stepwise entry to test for the contribution of each acoustic parameter to separating perceptually determined call types (the dependent categorical variable: grunt, squeal, trumpet, or grunt‐squeal). The Bayesian information criterion (BIC) was used to determine the best‐fitting model. BIC was preferred over the Akaike information criterion as it more severely penalizes an increase in the number of parameters obtained in the final model and is considered more appropriate for inferential questions like those addressed here.

Following application of the MLR, a hierarchical cluster analysis (HCA) was conducted using the parameters retained in the best MLR model, to compare the perceptual classification to a classification produced by a more objective, ‘hands‐off’ approach. The HCA was performed using Ward's method (Ward 1963), with Euclidean distances (Szekely & Rizzo 2005), no normalization (as the data were already z‐score transformed), and a clustering range of 2–4 clusters. To evaluate how the resulting clusters compared with the perceptual classification, the Hubert‐Arabie adjusted Rand Index was calculated following Warrens (2008). This procedure assesses the similarities and differences between two classifications, generating a contingency table from which Cohen's Kappa can be computed.

For cross‐validation purposes, the same MLR followed by the HCA procedure was also conducted on a subsample of the data set, with an equal number of calls in each call type (53, the minimum number of cases in one group; in groups with an initial sample size of more than 53, cases were randomly selected). The only difference in this cross‐validation is that no weighting needed to be applied to the calls in the data set because the selected groups were equal in size.

## Results

### Perceptual Classification

**Figure 2:**
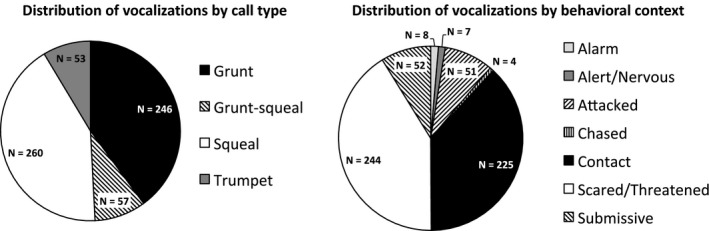
The distribution of recorded and analyzed vocalizations, as a function of call types and of behavioral contexts.

616 high‐quality calls were used to establish a perceptual classification scheme for the wild boar vocalizations recorded, based on previous research, and leading to the designation of four different classes (see Methods): grunts, squeals, trumpets, and grunt‐squeals (see Fig. 1). Grunts and squeals were the most common call types, while contact and threat situations were the most common contexts in which vocalizations were produced (see Fig. 2, Figures S1 and S2; Tables S2 and S3).

### Multinomial Logistic Regressions

**Figure 3:**
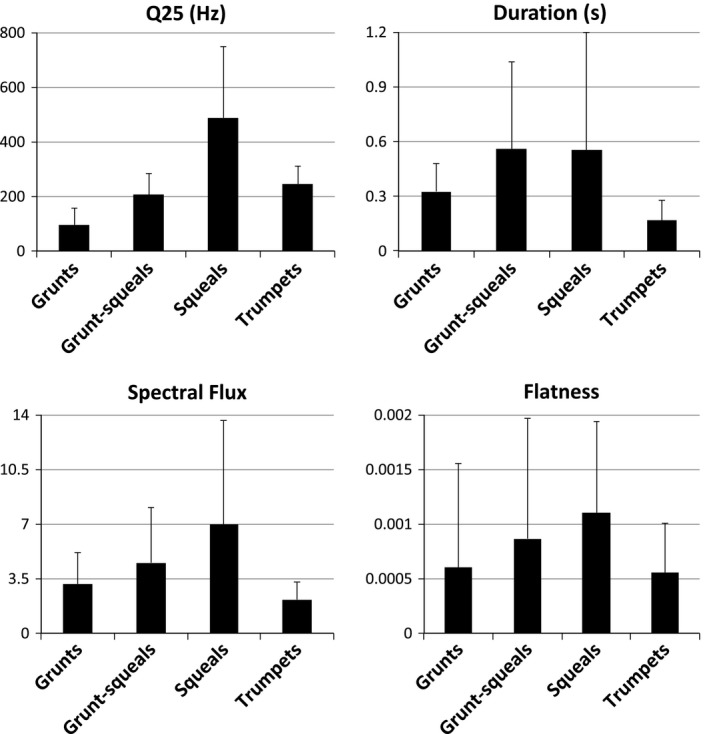
For each acoustical parameter retained from our MLR classification, mean values (±SD) are displayed for the four call types identified.

In the ‘DF regression’ model, the best fit was obtained for the final model including the following 6 predictor variables, sorted from most to least significant: DF, DUR, SF, FLAT, MDDF, and DSF (*χ* ^2^(18) = 968.91, p < 0.001). This final model had the lowest BIC and shows an overall classification agreement of 77.6% with our perceptual classification (meaning that in 77.6% of the cases, the MLR algorithm placed the calls within the same category as what was predicted by our perceptual classification; see details Table S4). In the ‘Q25 regression’ model, the best fit was obtained for the final model including the following 4 predictor variables, sorted from most to least significant: Q25, DUR, SF, FLAT (*χ* ^2^(12) = 1018.58, p <* *0.001). This final model had the lowest BIC and shows an overall classification agreement of 80.2% with our perceptual classification (Table S4). This second model was judged to be superior to the first model because it showed a higher classification accuracy (improving the classification of squeals and trumpets (respectively, by 5.8% and 5.6%), while losing only 0.8% of correct classification for grunts; this is likely due to the fact that DF is more susceptible to artifacts than Q25, which accounts for a range of frequencies rather than a single value), while using fewer predictor variables and with a higher *χ* ^2^. Additionally, the agreement percentage between this model and the perceptual classification was significantly above chance (25% as data were weighted; *Z* = 31.66, p < 0.001; calculated following (Titus et al. 1984). These four acoustic parameters—Q25, DUR, SF, and FLAT—were thus retained for subsequent cluster analysis (Fig. 3).

Cross‐validation of this second model based on a randomly selected subsample with equal numbers of calls in each category (53 for each call type, resulting in a total of 212 calls) showed very similar results. Starting with the same set of 10 variables, the final model built on this subsample again showed the best fit for Q25, DUR, SF, FLAT (*χ* ^2^(12) = 353.76, p <* *0.001), with an overall classification agreement of 81.6% with our perceptual classification, and calls distributed between categories in similar proportions to the full sample model (see Table S4).

### Hierarchical Cluster Analysis

The hierarchical cluster analysis clustered calls into 2, 3, and 4 clusters. The agreement with the perceptual classification is represented by Cohen's κ values, which were 0.33, 0.25, and 0.23 for 2, 3, and 4 clusters, respectively. Even though these values indicated a modest amount of agreement between the initial perceptual classification and the HCA classification, all were highly significant, suggesting that the observed levels of agreement were not accidental (2 clusters: z (κ/σ = 128.74, p <* *0.001; 3 clusters: z (κ/σ) = 105.51, p < 0.001; 4 clusters: z (κ/σ) = 100.84, p <* *0.001). The best agreement (suggested by the highest Cohen's κ) between the initial and the HCA classification was obtained for the 2‐cluster solution. For this solution, the first cluster included 84.2% of the squeals, 14.6% of the grunts, 13.2% of the trumpets, and 57.9% of the grunt‐squeals, whereas the second cluster included 15.8% of the squeals, 85.4% of the grunts, 86.8% of the trumpets, and 42.1% of the grunt‐squeals. Figure 3 and Figure S3 support this cluster analysis, separating in particular grunts and squeals, with grunt‐squeals placed as intermediate between those.

## Discussion

### Proposed Classification and Graded vs. Continuous Vocal Signaling

In this study, we present and statistically assess a four‐call‐type classification scheme for the wild boar vocal repertoire, based on a homogenous sample of high‐quality recordings of vocalizations from a semi‐free ranging population. We used information from previous studies examining domestic pig (Kiley 1972) and wild boar (Klingholz et al. 1979) repertoires as a starting point but avoided biases potentially introduced by human perception (Range & Fischer 2004), that is, the knowledge of the behavioral context in which vocalizations were produced. Like other recent analyses of domestic pig vocalizations (Tallet et al. 2013), we made an initial perceptual classification based on the existing literature and on the vocalizations alone and then verified our classification scheme using statistical methods and objective acoustic properties. A comparison of the MLR classification with our perceptual classification performed on the same data set showed 80.2% agreement (Table S4), suggesting that the four perceptually defined call types (grunts, squeals, grunt‐squeals, and trumpets) correspond to objectively measurable differences in wild boar vocalizations**.** In addition, the MLR classification identified four acoustic parameters, quartile Q25, duration, spectral flux, and spectral flatness (respectively, Q25, DUR, SF, and FLAT) as the best parameters to discriminate among the four call categories.

However, our results leave space for a certain degree of flexibility in the way we can categorize call types, similarly to previous findings in domestic pigs (Kiley 1972; Tallet et al. 2013) and suggestions for wild boars (Klingholz et al. 1979). On the one hand, the classification we propose was robust when Q25, DUR, SF, and FLAT were used in combination, and the separation between call types appeared distinct for instance when considering the opposition between grunts and squeals (see Fig. 3 and Fig S3) or the highly distinguishable trumpet calls (94.3% agreement between the perceptual and the MLR classification, see Table S4). On the other hand, considerable overlap was observed when the average values of Q25, DUR, SF, and FLAT were compared across call types (see Fig. 3), indicating transitions from one call type to another, that is, the grunt‐squeals representing an acoustic intermediate between grunts and squeals. This interpretation is supported by the fact that grunt‐squeals were less well classified in the MLR model (64.9%) and mixed between the two clusters in the HCA (57.9% of grunt‐squeals pertaining to the cluster containing most of the squeals and 42.1% in the cluster containing most of the grunts; see also Fig. 3 and Fig S3).

More generally, these results raise the question of acoustic continuity and illustrate the ambiguity that can emerge from interpreting a species’ vocal production and/or perception in a categorical manner, a topic which has been widely discussed (e.g., Marler 1975, 1976; Morton 1982; Cheney & Seyfarth 1990; Volodina 2000; Boisseau 2005; Stoeger‐Horwath et al. 2007; Nair et al. 2009; Fitch 2010). Marler (1975) proposed that the evolution of graded and discrete signals depends on a species’ habitat and social structure. In particular, graded acoustic signals have been suggested to prevail in species living in open habitats with frequent close range interactions occurring between conspecifics (Marler 1976; Keenan et al. 2013; Manser et al. 2014). Accordingly to this hypothesis, the highly social lifestyle of wild boars (Meynhardt 1990) predicts continuous acoustic variation, while the fact that they often occupy dense, closed habitats (Wilson & Mittermeier 2011) predicts discrete call types. We found evidence supporting both of Marler's predictions in our vocal repertoire analysis, including continuous and discrete components. One important caveat common to all study species, however, is that humans do not necessarily perceive sounds like the animals themselves (Range & Fischer 2004), and while an acoustic continuum is present at the production level, conspecific receivers may perceive discrete calls (Byrne 1982; Slocombe et al. 2009). Here, we suggest that avoiding the consideration of contextual framework may enhance objectivity and reduce biases while establishing distinct call categories, but that one should not neglect the potential existence of vocal intermediates, both in terms of the acoustic structure on the production side and of the animal's perception**.** The only way to thoroughly investigate a species vocal communication at this level is to run playback experiments testing the subtle acoustic variations, in particular if conducted in combination with signal processing and re‐synthesis techniques (Reby et al. 2005; Fitch & Fritz 2006; Charlton et al. 2012; Garcia et al. 2013).

### Animal Welfare and Domestication

From a more applied and practical perspective, the fact that grunt‐squeals have an intermediate acoustical structure between grunts and squeals may also have implications for the field of animal welfare. Inspection of the relation of grunt‐squeals to behavioral context shows that 96.6% of grunt‐squeals occurred in situations that were likely experienced by the animals as negatively valenced (being scared/threatened, requiring submission, attacked), although they rarely occurred in situations likely to evoke pain (Tallet et al. 2013), as being attacked only represented 1.8% of these cases (see Fig S2 and Table S3). Instead, they were more likely to be produced at intermediate levels of negativity (scared/threatened: 82.5%; submissive: 12.3%; see Table S3). Thus, as suggested in domestic pigs (Kiley 1972; Schrader & Todt 1998), in addition to representing a transition between grunts and squeals acoustic structures, grunt‐squeals may also represent an intermediate expression of negative emotional valence. As grunt‐squeals in this study were classified with 64.9% accuracy by the MLR model, we provide some support for the possibility of reliable automatic identification and therefore the opportunity to alleviate suffering before animals reach higher/maximal levels of discomfort and/or suffering, such as described in various studies (Weary et al. 1998; Taylor et al. 2001; Marx et al. 2003; Hillmann et al. 2004; Puppe et al. 2005; Düpjan et al. 2008; Leidig et al. 2009). These results advocate the overall importance of acoustic studies in the field of animal welfare, especially those investigating the vocal correlates of emotion (Briefer 2012) for improving the care given to farm animals (see for example, von Borell et al. (2009)).

From a more evolutionary point of view, the question of the effect of domestication on vocal production arises from our study in comparison with the domestic pig. The trumpet reported in this study does not seem to exist in the domestic pig repertoire (Tallet et al. 2013). Like most grunts, trumpets were typically used as contact calls. However, trumpets comprise different frequency contents than grunts. The use of contact calls with different frequency contents in wild boars could function as an adaptation to communicate in diverse acoustic environments (low and high frequencies show different propagation properties in different habitats; (Marten & Marler 1977) and/or may modulate the ability of conspecifics/predators to localize the vocalizer (the directionality of sound varies depending on frequency content; Richards & Wiley 1980). While the data necessary to test these possibilities are not presently available, they might explain why domestic pigs, which are not under the same selective pressures as wild boars, do not seem to produce trumpets.

Potential effects of domestication on vocal behavior are also apparent in other species. Adult dogs, for example, show increased barking compared to adult wolves (Yin 2002) and adult domestic cats, unlike undomesticated cats, often continue to produce vocalizations typically only made by kittens, such as purring or meowing (Bradshaw & Cameron‐Beaumont 2000). Thus, farm animals and pets represent ideal targets for investigating the conservation of emotional vocal indicators throughout evolution or the changes in repertoire use that a species could undergo, because of the modification of different factors such as basic needs, social interactions, or environment quality. Further research is greatly needed to obtain a clear understanding of the selective forces affecting vocal communication that are generated by the domestication process.

### Limits and Prospects

Our conclusions are subject to some limitations. First, we do not claim to have exhaustively assessed the complete wild boar repertoire. Some specific call types may have been missed, for example, no male advertisement calls (Klingholz et al. 1979) were recorded despite having been observed on previous occasions at our field site (MG, personal observation). Second, we may have overestimated the apparent continuity of the acoustic signals in our analysis due to a methodological issue generally found in current acoustic research: the fact that each recording necessarily contains some residual background noise may mask some information and might increase the apparent similarity between sounds that would appear more different under perfect conditions. Apparent gradation could also be caused by the different frequency responses of the microphone depending on the recording angle, even though we believe this effect is negligible due to the high variability of the calls.

Finally, our study species may employ acoustic parameters that are not easily perceived by humans (Range & Fischer 2004) or that we did not measure. In particular, as periodicity and fundamental frequency could not be consistently measured for all calls, they were not examined here, despite being used to differentiate call types in other species (Volodina 2000; Leong et al. 2003; Stoeger‐Horwath et al. 2007; Baotic et al. 2014). Another potentially important parameter, absolute sound intensity, was not included here because it would require an amplitude calibration based on measured emitter–microphone distances, which are difficult to measure accurately or consistently in the field (especially at night). However, perceptually, MG noted that a very low‐intensity level was typical of trumpet calls and those grunts produced as contact calls. It is not unlikely that amplitude, if measured correctly, could aid in call type discrimination.

One last acoustic parameter neglected here is formant frequencies, which could not be consistently extracted from the recorded vocalizations but is potentially important. Formant spacing and formant frequencies have indeed been shown to carry information regarding individuality (Reby et al. 2006; Vannoni & McElligott 2007) as well as size‐related attributes of the emitter in a variety of mammalian species (Fitch 1997; Reby & McComb 2003; Sanvito et al. 2007; Vannoni & McElligott 2008; Charlton et al. 2009, 2011).

## Conclusions

The repertoire presented here is the most objective comprehensive analysis to date of the common wild boar vocalizations and should thus serve as a solid foundation for subsequent analyses and extensions. The issues we raised concerning the search for objectivity and acoustic continuity may benefit other studies investigating animal vocal repertoires. Additionally, the relationships we have emphasized to animal welfare and the effect of domestication should be taken into consideration to introduce new approaches, for both applied and evolution‐oriented acoustic research**.**

## Supporting information


**Table S1.** Parameters extracted from audio files and used in the statistical analysis, together with their definitions (see also Gingras & Fitch 2013).
**Table S2.** Proportion of call types associated with each behavioral context.
**Table S3.** Proportion of behavioral contexts associated with each call type.
**Table S4.** Classification agreement between perceptual classification and MLR models.
**Figure S1.** Proportion of call types associated with each behavioral context (calls for which the context of emission is unknown are not represented here; see Table S2 for detailed values).
**Figure S2.** Proportion of behavioral contexts associated with each call type (calls for which the context of emission is unknown are not represented; see Table S3 for detailed values).
**Figure S3.** The two principal components (PCA1 and PCA2) resulting from a PCA run on the four variables retained from the best MLR model (Q25, DUR, SF and FLAT), illustrating grunt‐‐‐squeals' acoustic structure as an intermediate between grunts and squeals.
