# Supplementary material for: Structural Classification of Wild Boar (Sus scrofa) Vocalizations
Source: Ethology. 2016 Feb 16;122(4):329–42. doi: 10.1111/eth.12472 (PMC4793927; doi:10.1111/eth.12472)
Supplement: Supplementary file 1 — Table S1. Parameters extracted from audio files and used in the statistical analysis, together with their definitions (see also Gingras & Fitch 2013). Table S2. Proportion of call types associated with each behavioral context. Table S3. Proportion of behavioral contexts associated with each call type. Table S4. Classification agreement between perceptual classification and MLR models. Figure S1. Proportion of call types associated with each behavioral context (calls for which the context of emission is unknown are not represented here; see Table S2 for detailed values). Figure S2. Proportion of behavioral contexts associated with each call type (calls for which the context of emission is unknown are not represented; see Table S3 for detailed values). Figure S3. The two principal components (PCA1 and PCA2) resulting from a PCA run on the four variables retained from the best MLR model (Q25, DUR, SF and FLAT), illustrating grunt‐‐‐squeals' acoustic structure as an intermediate between grunts and squeals. [file ETH-122-329-s001.pdf]

Parameter extracted	Definition of the parameter extracted
**1. Duration (DUR)**	Duration of the vocalization, in seconds
**2. Quartile Q25 (Q25)**	Frequency in the spectrum below which 25% of the energy in the signal is comprised, in hertz
**3. Quartile Q50 (Q50)**	Frequency in the spectrum below which 50% of the energy in the signal is comprised, in hertz
**4. Quartile Q75 (Q75)**	Frequency in the spectrum below which 75% of the energy in the signal is comprised, in hertz
**5. Mean dominant frequency (DF)**	Point of maximal amplitude along the frequency spectrum (here mean is calculated from DF obtained in each analysis window), in hertz
**6. Mean differential of the dominant frequency (DDF)**	Mean difference between points of maximal amplitude along the frequency spectrum from 2 consecutive analysis windows, in hertz
**7. Maximum differential of the dominant frequency (MDDF)**	Maximum difference between points of maximal amplitude along the frequency spectrum from 2 consecutive analysis windows, in hertz
**8. Standard deviation of the dominant frequency (STDF)**	Standard deviation of the points of maximal amplitude along the frequency spectrum (here obtained from all analysis windows within a sound file), in hertz
**9. Spectral centroid (SC)**	Weighted mean by amplitude of the frequencies present in the audio signal (here mean is calculated from SC obtained in each analysis window), in hertz
**10. Spectral entropy (SE)**	Measurement assessing the regularity of the spectrum by returning the relative Shannon entropy of the input spectrum, no unit
**11. Coefficient of variation of the root-mean-square of the amplitude (CVA)**	Standard deviation of the root-mean-square of the amplitude divided by the true mean, no unit
**12 Mean differential of the normalized root-mean-square (DRMS)**	Mean difference between the normalized root-mean-square from 2 consecutive analysis windows, no unit
**13. Standard deviation of the normalized root-mean-square (STRMS)**	Standard deviation of the normalized root-mean-square (here obtained from all analysis windows within a sound file), no unit
**14. Spectral flatness (FLAT)**	Ratio between the geometric mean and the arithmetic mean (here mean is calculated from FLAT obtained in each analysis window); indicates whether a frequency spectrum is smooth ("flat") or spiky (contains definite peaks), no unit
**15. Spectralflux (SF)**	Distance between the spectra of successive frames (here Euclidean distances were use and mean is calculated from SF obtained in each analysis window), no unit
**16. Mean differential of the spectral flux (DSF)**	Mean difference of the distances between the spectra of successive frames, no unit
**17. Maximum differential of te spectral flux (MDSF)**	Maximum difference of the distance between the spectra of successive frames, no unit
**18. Standard deviation of the spectral flux (STSF)**	Standard deviation of the distance between the spectra of successive frames (here obtained from all analysis windows within a sound file), no unit
**19. Zero-crossing (ZC)**	Rate at which the amplitude of the audio signal changes from positive to negative and vice-versa (here mean is calculated from ZC obtained in each analysis window), no unit

ESM Table 1. Parameters extracted from audio files and used in the statistical analysis, together with their definitions (see also Gingras & Fitch 2013).

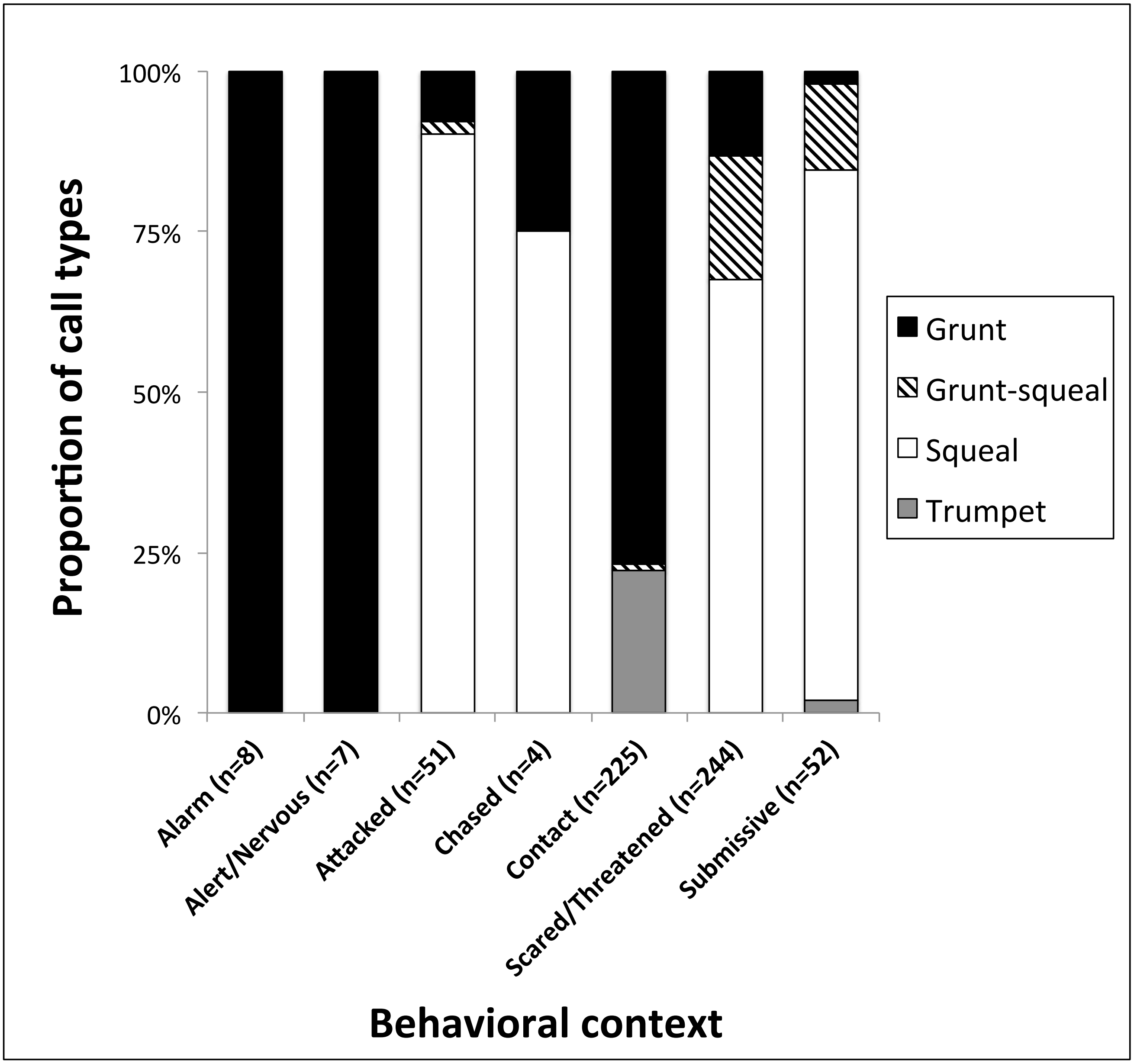

ESM Figure 1. Proportion of call types associated with each behavioral context (calls for which the context of emission is unknown are not represented here; see ESM Table 2 for detailed values).

Short description: All of the alarm and alert/nervous calls and most of the contact calls were grunts. The rest of the contact calls were mostly trumpets. The majority of the calls produced in negative contexts (e.g., scared/threatened, submission, attacked; see descriptions in Material and Methods; and see also McGlone 1985; Weng et al. 1998; Oczak et al. 2013) were squeals and grunt-squeals.

Call type \ Behavioral context	Alarm	Alert/ Nervous	Attacked	Chased	Contact	Scared/ Threatened	Submissive	Unknown
**Grunt**	8 (100%)	7 (100%)	4 (7.8%)	1 (25%)	173 (76.9%)	32 (13.1%)	1 (1.9%)	20 (80%)
**Squeal**	0 (0%)	0 (0%)	46 (90.2%)	3 (75%)	0 (0%)	165 (67.6%)	43 (82.7%)	3 (12%)
**Trumpet**	0 (0%)	0 (0%)	0 (0%)	0 (0%)	50 (22.2%)	0 (0%)	1 (1.9%)	2 (8%)
**Grunt-squeal**	0 (0%)	0 (0%)	1 (2%)	0 (0%)	2 (0.9%)	47 (19.3%)	7 (13.5%)	0 (0%)
**Sum**	8 (100%)	7 (100%)	51 (100%)	4 (100%)	225 (100%)	244 (100%)	52 (100%)	25 (100%)

ESM Table 2. Proportion of call types associated with each behavioral context.

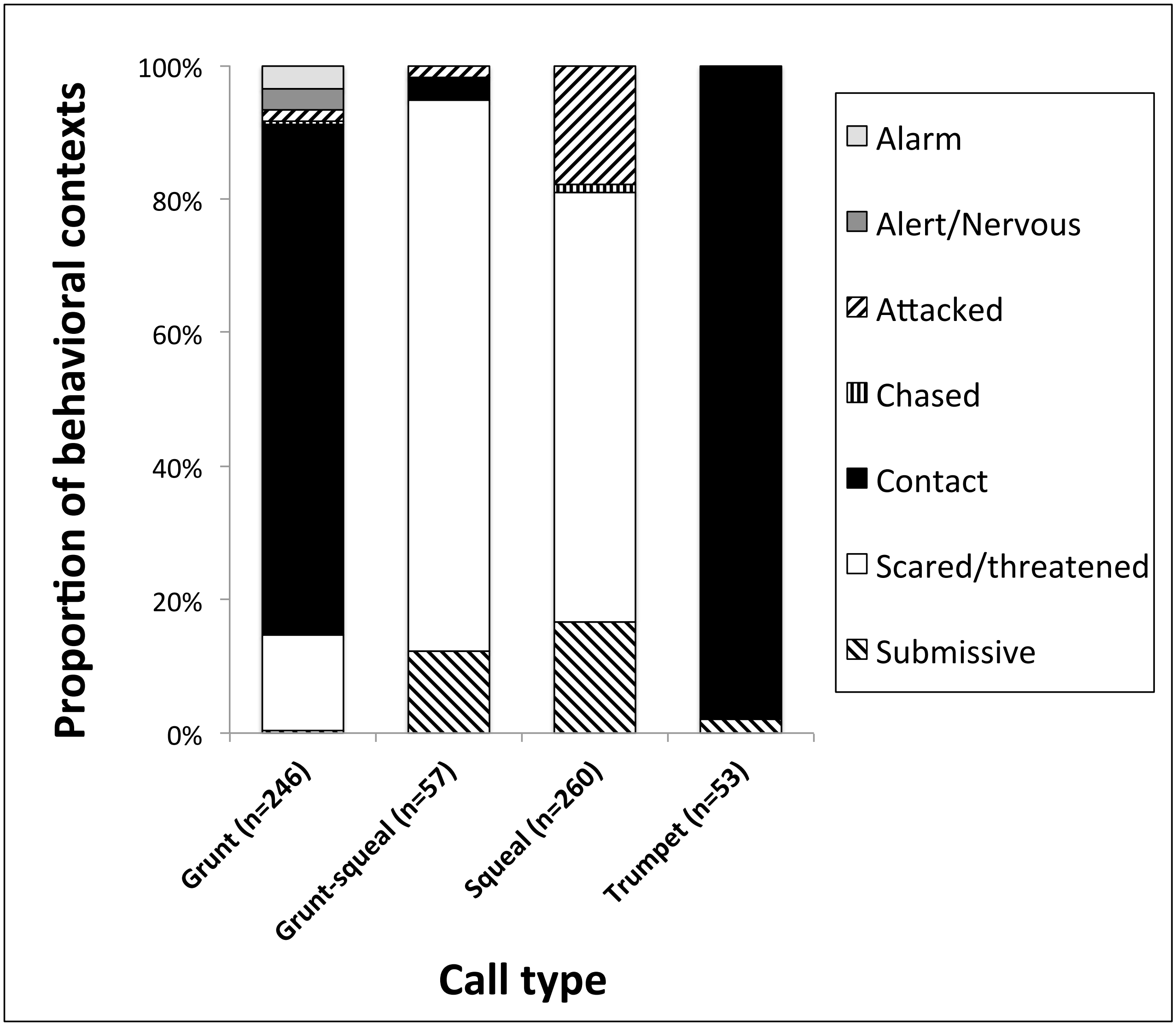

ESM Figure 2. Proportion of behavioral contexts associated with each call type (calls for which the context of emission is unknown are not represented; see ESM Table 3 for detailed values).

Short description: Whereas squeals were used across several negative contexts (being scared/threatened, during submission and when being attacked), most of the trumpets were contact calls and most of the grunt-squeals were scared/threatened calls. Grunts were used across all identified contexts, although predominantly as contact calls. Thus call types varied considerably in their context-specificity.

Behavioral context / Call type	Alarm	Alert/ Nervous	Attacked	Chased	Contact	Scared/ Threatened	Submissive	Unknown	Sum
**Grunt**	8 (3.3%)	7 (2.8%)	4 (1.6%)	1 (0.4%)	173 (70.3%)	32 (13%)	1 (0.4%)	20 (8.1%)	246 (100%)
**Squeal**	0 (0%)	0 (0%)	46 (17.7%)	3 (1.2%)	0 (0%)	165 (63.5%)	43 (16.5%)	3 (1.2%)	260 (100%)
**Trumpet**	0 (0%)	0 (0%)	0 (0%)	0 (0%)	50 (94.3%)	0 (0%)	1 (1.9%)	2 (3.8%)	53 (100%)
**Grunt-squeal**	0 (0%)	0 (0%)	1 (1.8%)	0 (0%)	2 (3.5%)	47 (82.5%)	7 (12.3%)	0 (0%)	57 (100%)

ESM Table 3. Proportion of behavioral contexts associated with each call type.

MLR model / Call type	DF Regression (N = 616)	Q25 Regression (N = 616)	Cross-validation (N = 212)
Squeals	66.9 %	72.7 %	73.6 %
Grunts	89.8 %	89 %	88.7 %
Grunt-squeals	64.9 %	64.9 %	69.8 %
Trumpets	88.7 %	94.3 %	94.3 %
**Overall**	**77.6 %**	**80.2 %**	**81.6 %**

ESM Table 4. Classification agreement between perceptual classification and MLR models

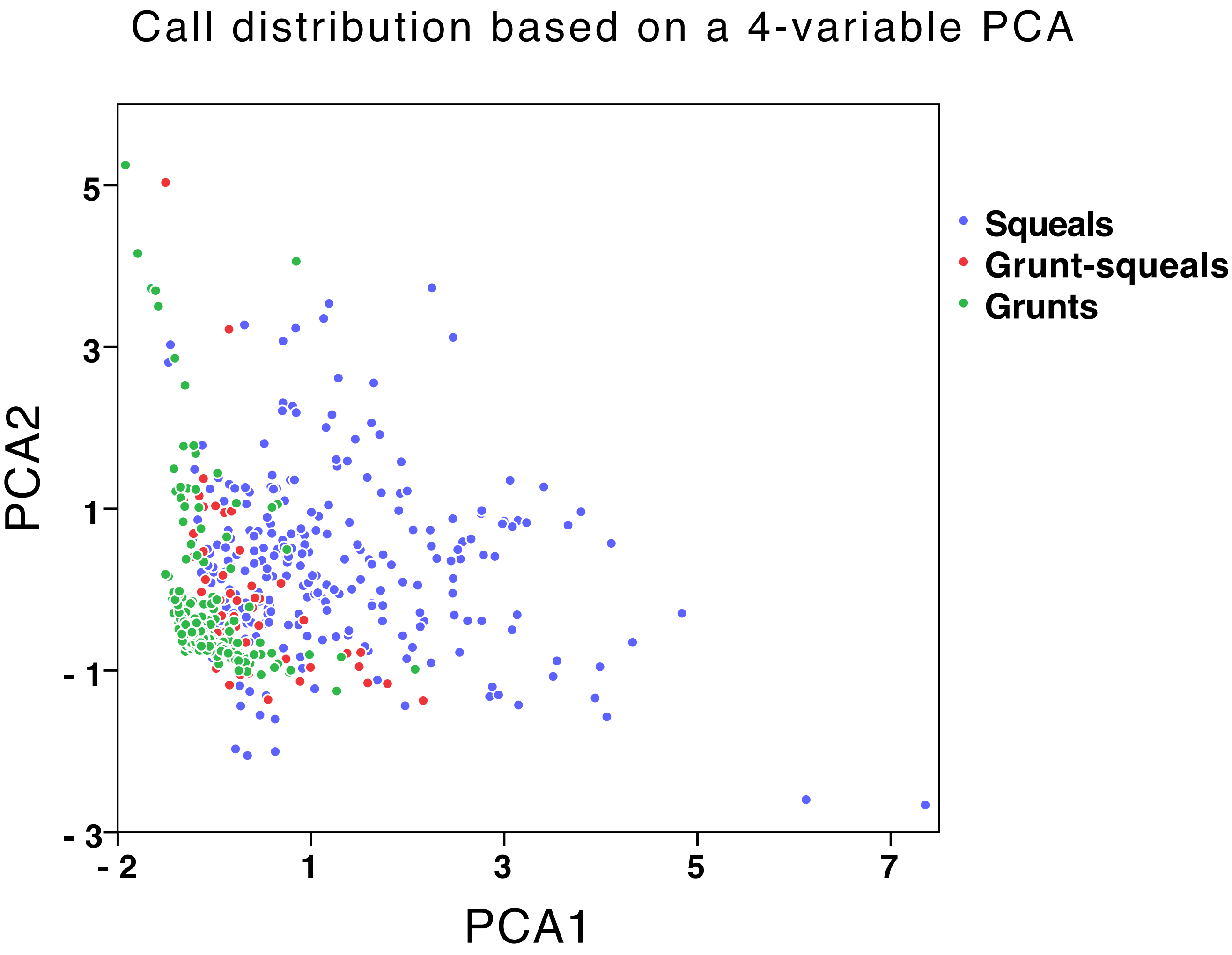

ESM Figure 3. The two principal components (PCA1 and PCA2) resulting from a PCA run on the four variables retained from the best MLR model (Q25, DUR, SF and FLAT), illustrating grunt-squeals' acoustic structure as an intermediate between grunts and squeals.

References:

Gingras, B. & Fitch, W. T. 2013: A three-parameter model for classifying anurans into four genera based on advertisement calls. J. Acoust. Soc. Am. **133**, 547-559.

McGlone, J. J. 1985: A quantitative ethogram of aggressive and submissive behaviors in recently regrouped pigs. J. Anim. Sci. **61**, 559-565.

Weng, R. C., Edwards, S. A. & English, P. R. 1998: Behaviour, social interactions and lesion scores of group-housed sows in relation to floor space allowance. Appl. Anim. Behav. Sci. **59**, 307-316.

Oczak, M., Ismayilova, G., Costa, A., Viazzi, S., Sonoda, L. T., Fels, M., Bahr, C., Hartung, J., Guarino, M., Berckmans, D. & Vranken, E. 2013: Analysis of aggressive behaviours of pigs by automatic video recordings. Comput. Electron. Agric. **99**, 209-217.
